# Turing miRNA into infinite coordination supermolecule: a general and enabling nanoengineering strategy for resurrecting nuclear acid therapeutics

**DOI:** 10.1186/s12951-021-01212-9

**Published:** 2022-01-04

**Authors:** Liya Li, Wangxiao He, Weiming You, Jin Yan, Wenjia Liu

**Affiliations:** 1 Institute for Stem Cell & Regenerative Medicine, The Second Affiliated Hospital of Xi’an Jiaotong University, Xi’an, 710004 China; 2 Department of Medical Oncology and Department of Talent Highland, The First Affiliated Hospital of Xi’an Jiaotong University, Xi’an, 710061 People’s Republic of China; 3 National & Local Joint Engineering Research Center of Biodiagnosis and Biotherapy, The Second Affiliated Hospital of Xi’an Jiaotong University, Xi’an, 710004 People’s Republic of China

**Keywords:** miRNA clinical translation, Nuclear acid therapeutics, Nanoengineering, Infinite covalent polymer, Anti-cancer therapeutics

## Abstract

**Background:**

Clinical translation of therapeutic nuclear acid, particularly those targeting tumor progression, has been hampered by the intrinsic weaknesses of nuclear acid therapeutic including poor systemic stability, rapid clearance, low membrane permeability and lack of targeting ability. Small nuclear acid engineered into carrier-free nanodrugs with structural stability and disease targeting may be viable to overcome pharmaceutical obstacles of nuclear acid.

**Methods:**

A general method through a mild and simple chemistry was established to convert therapeutic miRNA into an infinite Auric-sulfhydryl coordination supramolecular miRNA termed IacsRNA with near-spherical nanostructure, high colloid as well as anti-hydrolysis stability and low macrophage uptakes.

**Results:**

IacsRNA presented the increased half-life period in circulation and accumulation at tumor sites in comparison to normal miRNA. Moreover, Iacs-miR-30c showed no toxicity of viscera and sanguis system in the 5-time injection dosage of the treatment. More importantly, Iacs-miR-30c potently suppressed the Wnt signaling pathway in vitro and in vivo, and effectively sensitized both potency of 5-Fu in PDX model of colon cancer and Anti-PD1 in B16F10 homograft model of melanoma.

**Conclusion:**

Collectively, this work amply confirmed the design of IacsRNA as a general and viable strategy of nano-pharmaceutic to concert flimsy therapeutic miRNA into potential drugs. Considering from a broader perspective, the miRNA-initiated infinite coordination self-assembly strategy has distinct advantages in resurrecting nuclear acid therapeutics, probably bringing new inspiration to RNA-derived therapeutics of a great variety of human diseases including cancer.

**Graphical Abstract:**

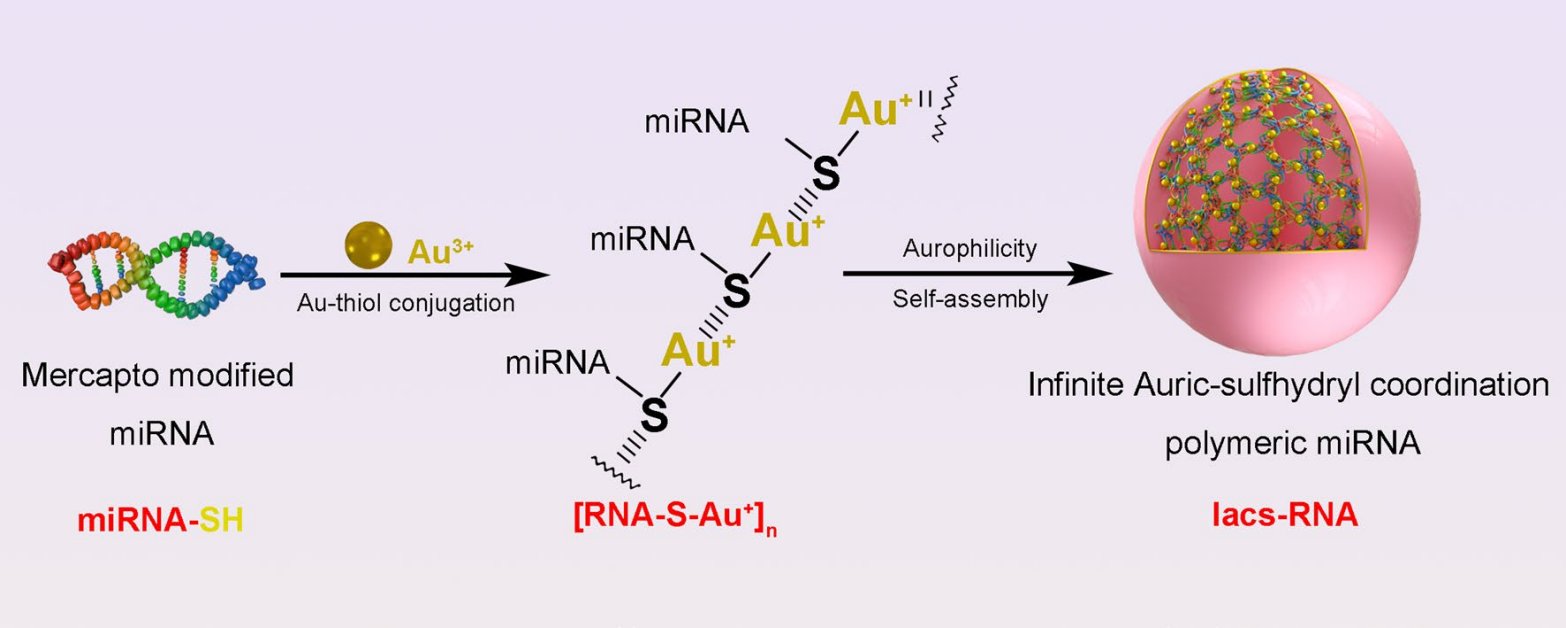

**Supplementary Information:**

The online version contains supplementary material available at 10.1186/s12951-021-01212-9.

## Introduction

MicroRNAs (miRNA), a series of 22 nucleotide-long and noncoding RNA molecules, regulate gene expression and a range of biological functions involving in the pathogenesis of a wide variety of diseases in especial of cancer [1]. In the cytoplasm, miRNAs recognize a complimentary mRNA sequence, and subsequently induce its post-translation degradation and silencing, offering great promise to create novel therapeutic approaches at transcriptional level towards down-regulating abnormally elevated pathogenesis-related proteins [2, 3]. Although a growing number of miRNAs modulating disease processes have been identifying, their clinical translation, particularly those targeting tumor progression, has been hampered by the intrinsic weaknesses of nuclear acid therapeutic including poor systemic stability, rapid clearance, low membrane permeability and lack of targeting ability [1, 4].

To alleviate these technical hurdles, various elaborate methods for miRNA modification and/or delivery have been developed, and significant progress has been made in ways of improving pharmacological potentials of miRNAs therapeutics [1]. Though chemically modified miRNAs possess the resistant against nuclease and the subsequent prolonged half-life in the bloodstream, the off-target effects severely limit their clinical application presumably because of the broad but excrescent functionality of miRNA in the healthy organs and tissues [5, 6]. Besides, as for miRNAs delivery vehicles, the frequently-used used lipids, cationic polymers and biodegradable polymers always suffered from the rapid clearance by reticuloendothelial system (RES), in which liver and spleen macrophages eliminate these exogenous particles at a great lick from the circulation system [7, 8], resulting in the bereft or depressed potency of miRNAs therapeutics. Conceptually, novel and clinically viable strategies are needed to advance the clinical transformation of miRNAs.

Nanotechnology holds great promise in overcoming pharmacological weaknesses of miRNA [9, 10]. Nanocarrier-mediated oligonucleotide delivery is capable of protecting the cargo from nuclease degradation during the circulation, and promoting oligonucleotide internalizing into cytoplasm via endocytosis that can escape endosomal degradation [11–13]. In fact, nanoparticle-based drug delivery systems are particularly attractive in the treatment of solid tumors, as nanoparticles are capable of active crossing endothelial cells into the interstitial space of the neoplasm, thereby resulting in the preferential accumulation at tumor sites [14–16]. Despite increasing success in the development of tumor-targeting nanocarriers [17–21], the overwhelming majority of nano-medicine suffered from time/cost-consuming construction, low cargo loading and improper disruption in circulation [22, 23]. Therefore, it remains a challenge to explore miRNA-derived nanotherapeutics that simultaneously possess simple synthesis, high stability, satisfying loading and excellent performance.

Carrier-free nanodrugs provided an intriguing strategy to develop nanomedicine, by which drug themselves were assembled into well-defined nanostructures through self-assembly and/or coordination [24–27]. Infinite coordination polymers (ICP) is an emerging class of carrier-free nanodrug system, in which drugs connect with ions directly via coordination bonds [28, 29], yielding a series of advantages including adjustable high drug loading, compositional tunability, mild preparing condition, and infinitely controllable extension in spatial dimension [28, 29]. Based on these superiorities, we hypothesized that further assembled miRNA-derived ICP into infinite coordination supermolecules through intermolecular interaction can not only overcome the pharmacological weaknesses of nuclear acid therapeutics, but also possess excellent pharmaceutical superiority towards the optimized potency.

**Fig. 1:**
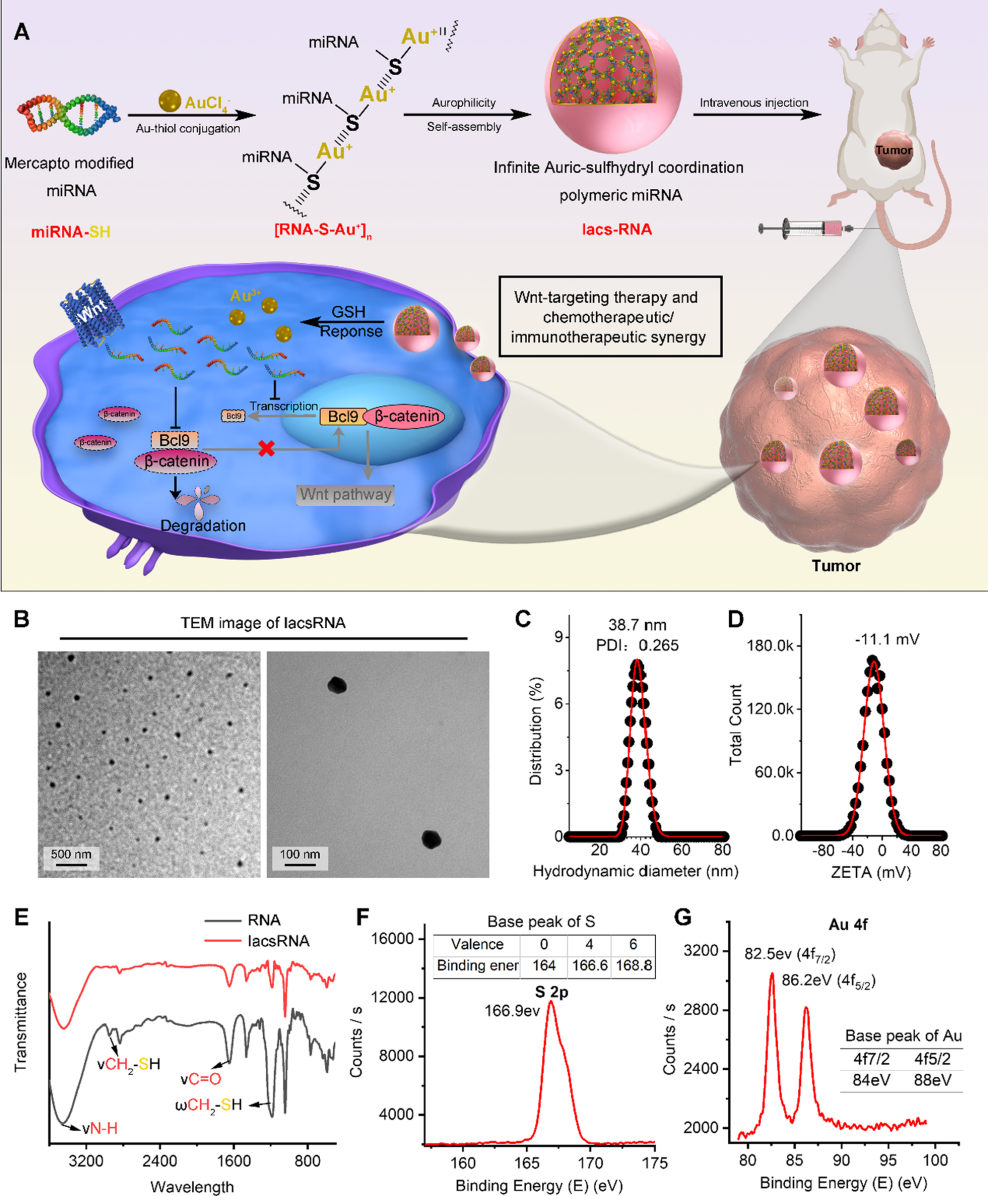
Fabrication and therapeutic mechanism of IascRNA. **A** Illustration of the synthesis and tumor response procedure of IacsRNA. **B** TEM images of IacsRNA. **C**, **D** Hydrodynamic diameter (**C**) and ZETA potential (**D**) of IacsRNA measured in PBS buffer at pH 7.4. **E** FT-IR spectra of IacsRNA and RNA. The band at 1200 cm^−1^ which was attributed to the stretching vibration of -SH. **F**, **G** S 2p XPS spectra (**F**) and Au 4f XPS spectra (**G**) of IacsRNA

Herein, we developed a general method to convert therapeutic miRNA into a stable and bioavailable infinite Auric-sulfhydryl coordination supramolecular miRNA (IacsRNA) by a mild and simple chemistry route. In this way, auric-mercapto-miRNA precursors can self-assembled into a spherical nanostructure driven by aurophilicity (Fig. 1A). As a proof of concept, miR-30c, a Wnt inhibitor was used to synthesized IacsRNA, termed Iacs-miR-30c (Fig. 1A). Responding to high concentrations of GSH in cancer cells, Iacs-miR-30c would depolymerize into free monomer, and target the expression of abnormally elevated β-catenin chaperonin, Bcl9 [30]. In this case, the nuclear translocation of β-catenin will be suppressed, resulting in the blockage of Wnt signaling pathway (Fig. 1A). By this way, Iacs-miR-30c overcome the pharmacological obstacles of miRNA and potently suppressed the Wnt signaling pathway in vitro and in vivo, but more than that, it effectively potentiated chemotherapy and immunotherapy in vivo. This work amply confirmed the design of IacsRNA as a general and viable strategy of nano-pharmaceutic to concert therapeutic miRNA into potential drugs, thereby reinvigorating efforts for discovering nuclear acid therapeutics in a great variety of human diseases including cancer.

## Results

### Design and synthesis of IacsRNA

For the construction of infinite Auric-sulfhydryl coordination polymeric miRNA (IacsRNA), an extra sulfydryl was introduced to the 5’ terminus of miRNA, and Au^3+^ in chloroauric acid was used to conjugate with the thiol to form a polymeric RNA-Au(I) compound termed [RNA-S–Au^+^]_n_ through infinite extension in 2D (Fig. 1A), where Au^1+^ ions bridge miRNA-SH by a bivalent Au^+^-SR coordination [19, 31–33]. Subsequently, driven by the aurophilicity among Au(I), a domino self-assembly of [RNA-S-Au^+^]_n_ occurred into a near-spherical supermolecule (IacsRNA) at small nano scale [18, 32], supporting by the transmission electron microscope (TEM) image of IacsRNA (Fig. 1B) and hydrodynamic diameter of IacsRNA around 38.7 nm with an acceptable polymey disperse index of 0.265 measured by dynamic light scattering analysis (Fig. 1C). Notably, because macrophages engulf particles ranging in diameter from 85 nm to 3.2 μm [34] and lymphocytes don’t engulf particles in diameter from 15 to 50 nm [35], 38.7 nm is an appropriate size for IacsRNA to escape from the phagocytose by macrophages and lymphocytes. Additionally, the surface charge of IacsRNA was -11.1 mV (Fig. 1D), and this electronegativity implied that IacsRNA would resist coronin and get a long half-life during circulation [36]. Moreover, UV–Vis (Additional file 1: Figure S1) and FT-IR (Fig. 1E) spectroscopy further confirmed the successful assembly of IacsRNA as evidenced by characteristic absorbance given by Mercapto modified RNA. The valence states of S and Au in IacsRNA were explore by X-ray photoelectron spectroscopy (XPS) analysis, in which S element presented + 4 valence in line with the expected molecular composition in [RNA-S-Au^+^]_n_ (Fig. 1F). Of note, the electronic energy signals given by Au in IacsRNA were low than the base electronic energy of Au atoms (Fig. 1G), presumably because of the electron migration from S to Au after conjugation, thereby further proving the construction of the infinite Auric-sulfhydryl coordination polymeric miRNA. Besides, the presence of phosphoric acid and ribonucleic groups on [RNA-S-Au^+^]n has the potential advantage of being pH-sensitive, which may adopt different sizes depending on the pH of HEPES solution. As shown in Additional file 1: Table S1, pH adjustment during the IacsRNA synthesis directly affected its hydrodynamic diameter. Collectively, these results demonstrated the successful conversion from miRNA to a near-spherical polymeric miRNA nanoparticle with controlled size through a simple and mild “one-pot” chemistry.

### Physicochemical and pharmaceutical properties of IacsRNA

**Fig. 2:**
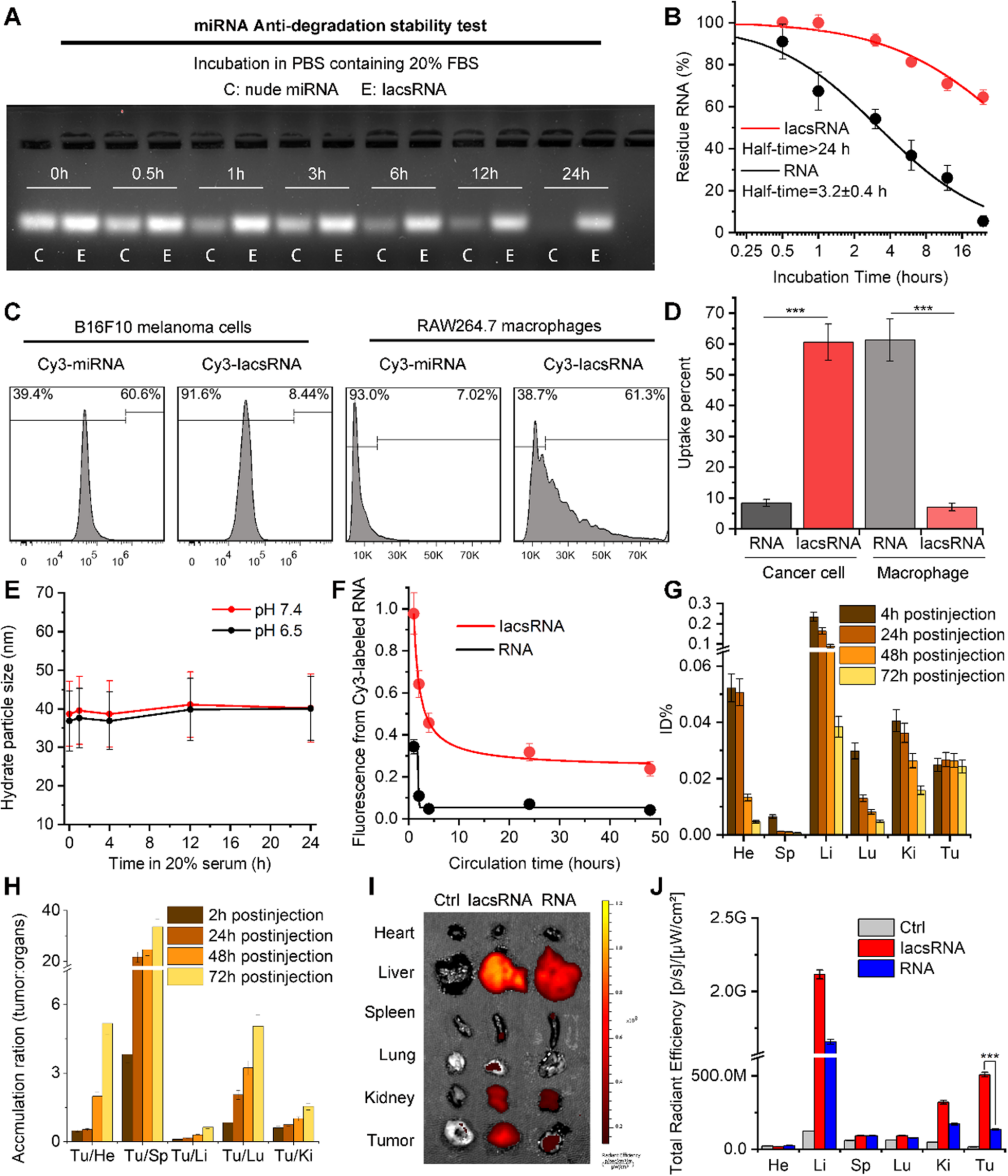
Physicochemical and pharmaceutical properties of IacsRNA. **A**, **B** Anti-degradation stability test (**A**) and quantitative analysis (**B**) of IacsRNA and nude miRNA by gel electrophoresis. **C**, **D** Flow cytometry analysis (**C**) and quantification (**D**) of different cells uptake of 1 µM Cy3-labelled miRNA and IacsRNA after 6 h incubations. (**E**) Colloidal stability of IacsRNA suspending in PBS containing 20% FBS at pH7.4 and 6.5 measured by DLS. **F** Blood-circulation curves of IacsRNA and RNA in healthy C57/B6 mice quantified by the fluorescence signal from the labeled Cy3 fluorescein in miRNA. **G** organ distribution of IacsRNA in BALB/c mice after systemic injection. Serial sacrifices were carried out at 2 h, 24 h, 48 h and 72 h after injection. Several organs/tissues, including heart, liver, spleen, lung, kidney and tumor were isolated to determine gold concentrations by ICP-MS. He, heart; Sp, spleen; Li, liver; Lu, lung; Ki, kidney; Tu, tumor. **H** Tumor-to-organ ratios for IacsRNA at 2 h, 24 h, 48 h and 72 h after injection. **I**, **J** Tissue distribution (**I**) and quantification (**J**) of IacsRNA. The fluorescence signal from the tumors and normal organ after IacsRNA intraperitoneal injection (200 µL, 1 mM Au) at 1 h. The data were presented as mean ± s.d. *, *p* < 0.05; **, *p* < 0.01; ***, *p* < 0.001

As our design, IacsRNA should be endowed with the resistance against endonuclease, because of the steric hindrance of near-spherical nanostructure against enzyme recognition and binding [37, 38]. To explore it, nude miRNA and its corresponding IacsRNA were incubated in sterile PBS buffer containing 20% foetal bovine serum (FBS), and the residue RNA were semi-quantified by agarose gel electrophoresis (Fig. 2A). In this case, IacsRNA obviously prolonged the half-time of RNA from 3.2 ± 0.4 h to over 24 h (Fig. 2B)., indicating that IacsRNA strategy can overcome the poor systemic stability—the major pharmaceutical obstacle—of nuclear acid therapeutics. Next, to explore the cellular internalization of IacsRNA, Sulfo-Cyanine3 (Cy3) was 3’-terminally conjugated for the cellular uptakes examination by flow cytometry analysis. As shown in Fig. 2C, D, IacsRNA (91.6%) showed much more internalization into B16F10 melanoma cells than miRNA without transfection (39.4%) after 6 h incubation. What's even more amazing is IacsRNA prevented the macrophages uptakes of miRNA (Fig. 2C, D), presumably because macrophages have the trend to engulf free nucleic acid other than particles smaller than 85 nm diameter [34, 39], providing a highly favorable profile for circulation. This result compelled us to explore the colloidal stability of IacsRNA that is another important influencing factor for blood circulation. As expected, both in pH 7.4 and pH 6.5, the incubation in PBS including 20% serum cannot alter the hydrodynamic diameter of IacsRNA (Fig. 2E), indicative of low coronin and high colloidal stability. As a result, compared to miRNA alone, IacsRNA significantly improved the circulation time in blood after systematic injection quantified by the fluorescence signal from the labeled Cy3 fluorescein in miRNA (Fig. 2F).

According to the enhanced permeability and retention (EPR) effect of nanoparticle, satisfactory circulation time always resulted in the promotional tumor accumulation. To verify it, we determined the biodistribution of IacsRNA in B16F10 homograft model of melanoma by inductively coupled plasma mass spectrometry (ICP-MS), which was used to quantify ^197^Au concentrations in tissues. After intravenous injection of 2 mg/Kg IacsRNA, a time-dependent tendency for tumor accumulation can be found in the biodistribution results (Fig. 1G) and calculating the accumulation ratios of tumor versus normal organs (Fig. 1H). To visually examine the different tumor accumulation between miRNA and IacsRNA, Cy3-labaled samples were intravenously injected into the tumor-bearing mice. At 6 h post-injection, ex vivo fluorescence imaging and quantification (Fig. 2I, J) revealed over 4-times tumor accumulation in Cys-IacsRNA-treated mice in comparison to Cys-miRNA-treated mice. Taken together, these results demonstrated that IacsRNA strategy overcome the intrinsic weaknesses of nuclear acid therapeutic including poor systemic stability, rapid clearance, low membrane permeability and lack of tumor targeting.

### The safety evaluation of IacsRNA in vivo

**Fig. 3:**
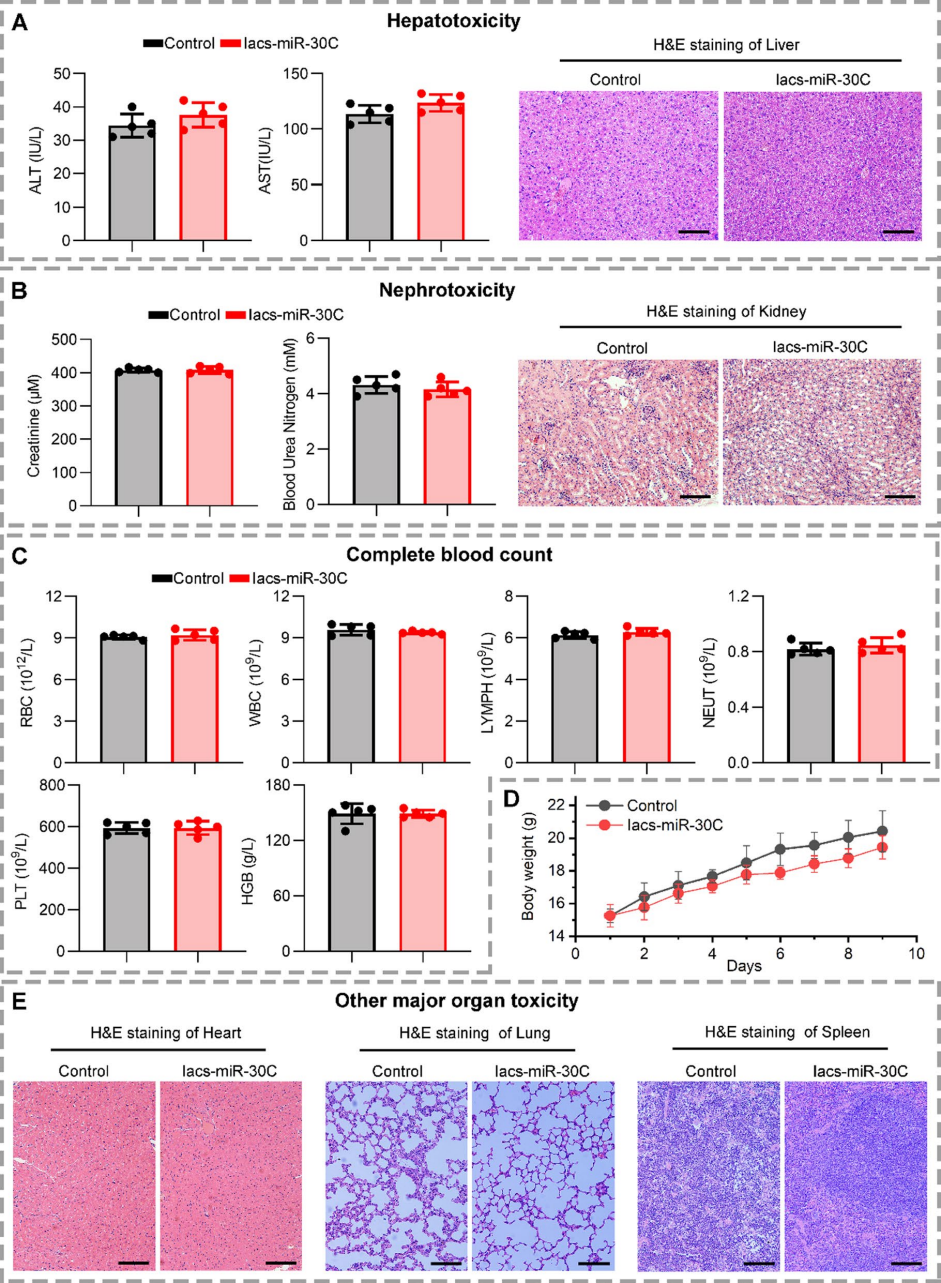
The biosafety evaluation of IacsRNA in vivo. **A** Hepatotoxicity testing of the IacsRNA measured by aspartate transaminase (ALT), alanine aminotransferase (AST), and pathological section of liver (scale bar: 100 μm). **B** Nephrotoxicity testing of the IacsRNA measured by blood urea nitrogen (BUN), creatinine (CRE) and pathological section of kidney (scale bar: 100 μm). **C** Analysis of red blood cell (RBC), white blood cell (WBC), lymphocyte (LYMPH), Neutrophil (NEUT), Platelets (PLT) and Hemoglobin (HGB) in mice blood with the indicated treatments. **D** Body weight of mice with the indicated treatments. **E** Toxicity testing of the IacsRNA measured by pathological section of heart, lung and spleen. The data were presented as mean ± s.d. *, *p* < 0.05

The superior performance of IacsRNA in pharmaceutical properties further compelled us to study its safety in vivo. To assess the safety of Iacs-miR-30C, healthy C57/BL6 mice were intravenously injected with normal saline (Control) or Iacs-miR-30C respectively every other day at a dosage of 10 mg/kg, which was 5-times the therapeutic dose (n = 5 per group). After 9 days of administration, no hepatotoxicity can be found as evidenced by the no difference of aspartate transaminase (ALT), alanine aminotransferase (AST) and pathological section of liver between Control and IacsRNA group (Fig. 3A). Besides, the 9-day administration have no effect on the blood urea nitrogen (BUN), creatinine (CREA) and Hematoxylin&Eosin (H&E) staining slices of kidney (Fig. 3B), indicative of the hardly any nephrotoxicity. In addition, no hemolysis, myelosuppression, anemia, leukopenia and thrombopenia was found after Iacs-miR-30C treatment (Fig. 3C). Moreover, compared with control group, Iacs-miR-30C treatment had almost no effect on body weight (Fig. 3C, D), which further supported the highly favorable biosafety profile of Iacs-miR-30C. Additionally, no pathological morphological changes can be found in the heart, lung, and spleen at Iacs-miR-30C-treated mice. Collectively, these data illustrated that Iacs-miR-30C is avirulent enough for clinical translation.

### IacsRNA potently suppressed Wnt/β-catenin pathway

**Fig. 4:**
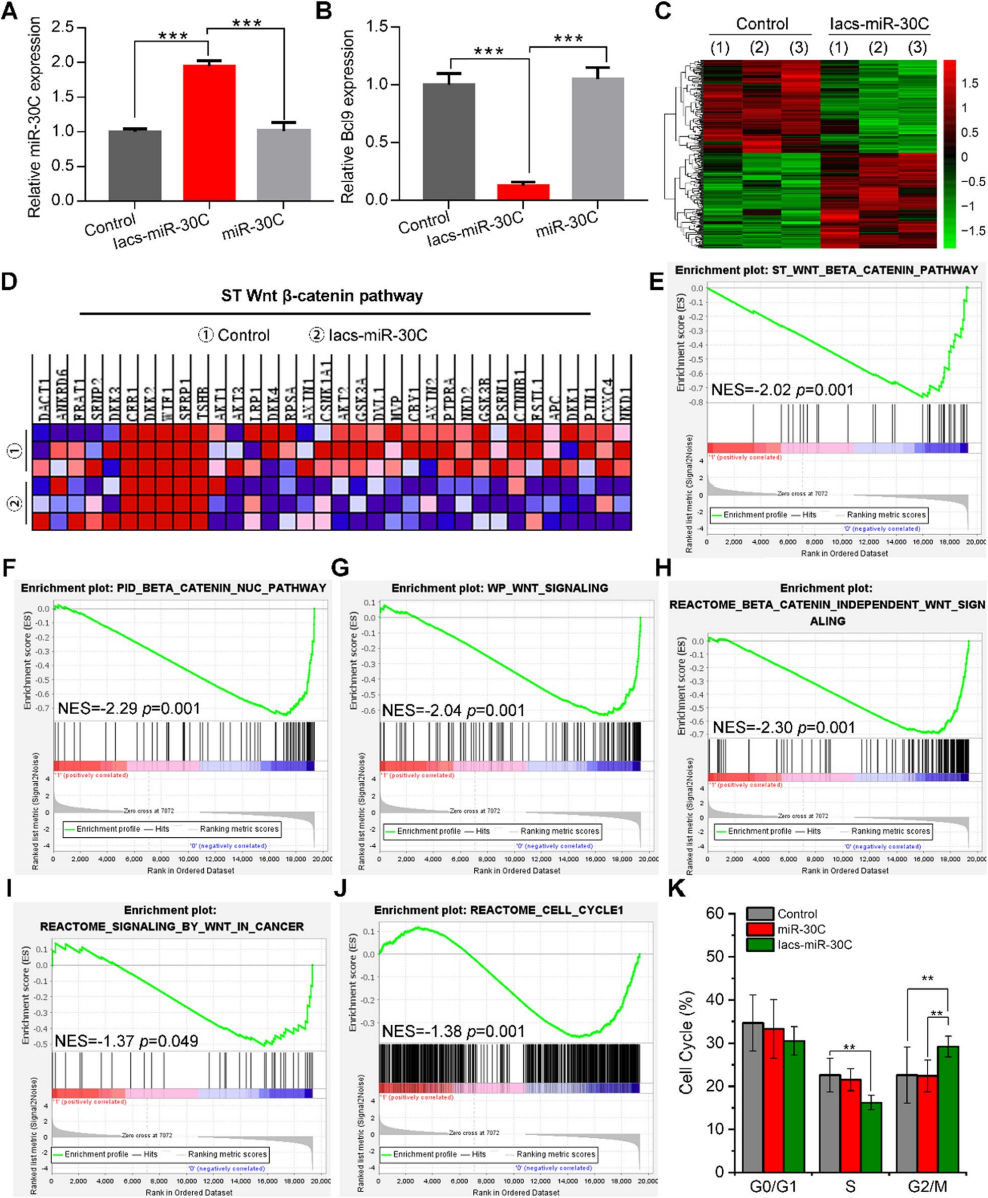
IacsRNA targeting Bcl9 inhibits Wnt pathway. **A**, **B** B16F10 cells were treated with IacsRNA and miR-30C for 24 h at the dosage of 50 nM, and qRT-PCR was performed to analyze the expressions of miR-30C (**A**) and Bcl9 (**B**). **C** Hierarchical clustering of genes differentially expressed in B16F10 cells after exposure to 50 nM IacsRNA for 24 h compared with miR-30C-treated cells (n = 3). **D** Hierarchical clustering of genes in Wnt/β-catenin signaling pathway. **E**–**J** Gene set enrichment analysis (GSEA) showing the Wnt/β-catenin pathway, β-catenin signaling pathway and cell cycle differentially expressed in response to IacsRNA. KEGG, Kyoto Encyclopedia of Genes and Genomes; PID, Pathway Interaction Database; Nes, normalized enrichment score. **K** Cell cycle was analyzed by flow cytometry. The data were presented as mean ± s.d. *, *p* < 0.05; **, *p* < 0.01

MiR-30c is known to inhibit oncogenic Wnt/β-catenin activation through suppressing the expression of Bcl9 [21]. To comparatively investigate the potency of Iacs-miR-30c and commercial miR-30c, B16F10 cell line- a kind of malignant and Wnt-hyperactive melanoma- was used to challenge their action via a 24-h incubation at the concentration of 50 nM. As expected, Iacs-miR-30c enhanced the accumulation of miR-30C in cells and suppressed the expression of Bcl9 potently, whereas carrier-free miR-30C showed no difference between PBS-mock-treated control (Fig. 4A, B). At a transcriptional level, Iacs-miR-30C triggered 360 differentially expressed genes after 24-h incubation on B16F10 cells as evidenced by RNA sequencing and subsequent clustering analysis (n = 3) (Fig. 4C). Gene set enrichment analysis (GSEA) exposed that suppression signatures were enriched in β-catenin gene sets in Iacs-miR-30C-treated cells compared to the mock-treated cells (Fig. 4D–F). Meanwhile, there was also a remarkably enrichment of suppression features presented in Wnt signaling pathway (Fig. 4G–I). As a consequence, Iacs-miR-30C induced the cell cycle arrest of cancer cells, as reflected in GSEA analysis in cell cycle, cell cycle checkpoints and cell cycle mitotic (Fig. 4J and Additional fie 1: Fig. S2). This resulted was proved again by the significantly decreased percentage of S phage of cells measured by the PI-staining-derived cell cycle analysis (Fig. 4K and Additional fie 1: Fig. S3). Together, above results demonstrated that IacsRNA strategy resurrected the action of miR-30c and potently suppressed Wnt/β-catenin pathway in vitro.

### IacsRNA potently suppressed Wnt/β-catenin pathway in vivo and augmented the potency of chemotherapy

**Fig. 5:**
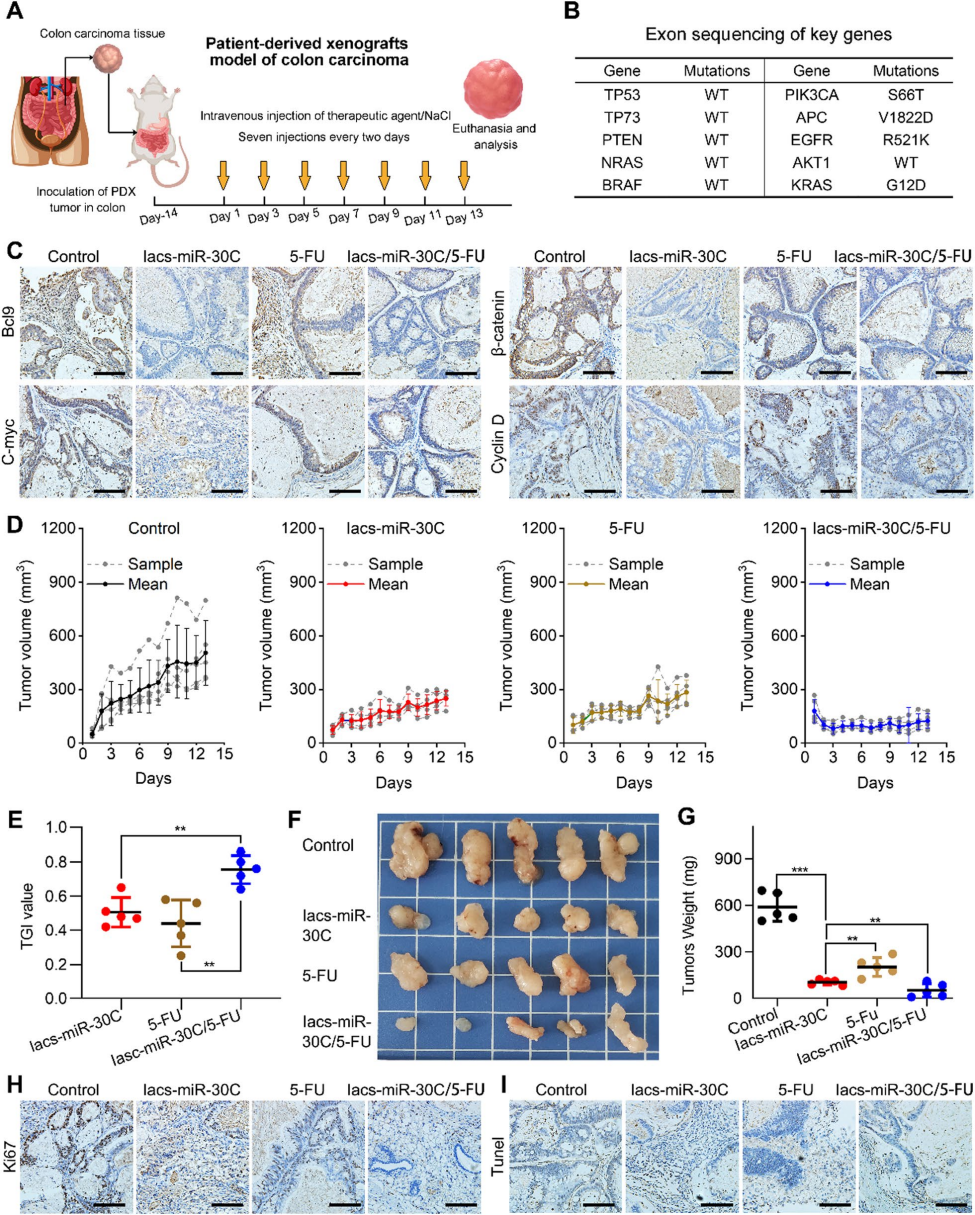
IacsRNA in vivo inhibited patient-derived tumor xenograft colon carcinoma in NOD/SCID mice. **A** Schematic diagram of PDX model construction. **B** Detection of tumor mutations by exon sequencing. WT stands for wild type. **C** Representative tumor sections after the indicated treatment staining by immunohistochemistry (scale bar: 100 μm). **D** Tumor growth curves in PDX mouse model mice with the indicated treatments (n = 5). **E** Tumor growth inhibition (TGI) with the indicated treatment at day 13. **F**, **G** Representative photographs (**F**) and weight of tumor (**G**) after the indicated treatments. **H**, **I** Ki67 (**H**) and TUNEL (**I**) staining in tumor sections from mice with the indicated treatments (scale bar: 100 μm). The data were presented as mean ± s.d. Statistical analysis was performed using t test, *, *p* < 0.05; **, *p* < 0.01; ***, *p* < 0.001

To further test the in vivo potency of Iacs-miR-30C, we comparatively explored its anti-cancer action with chemotherapeutic agent, and further investigated its synergistic sensitized effect with chemotherapy. Towards this end, a patient-derived-tumor-xenograft (PDX) model of colon carcinoma was established through subcutaneously transplanting surgically acquired colon tumor into the fossa iliaca of NOD/SCID mice (Fig. 5A). In this model, the acquired colon tumor was characterized by identifying missense mutated oncogenes, including PIK3CA (S66T), APC (V1822D), EGFR (R521K), KRAS (G12D) (Fig. 5B). Of note, V1822D mutation of APC suggested the hyper-action of Wnt signaling pathway, and G12D mutation of KRAS means the high malignancy and clinical incurability. When the tumor volume reached 100 ± 30 mm^3^, tumor-bearing mice were treated with NS (Control), Iacs-miR-30C, 5-FU (the first-line chemotherapy agent for colon cancer), or Iacs-miR-30C/5-Fu combo every other day for 13 days, involving intravenous injections at the dosage of 2 mg/kg Iacs-miR-30C, and/or of 5 mg/kg of 5-FU (Fig. 5A). At day 5, immunohistochemical staining image of tumors revealed that Iacs-miR-30C remarkably down-regulated Bcl9 and effectively suppressed the β-catenin/Wnt signaling cascade as support by the decreased protein level of Bcl9, β-catenin, and two Wnt- downstream proteins, C-myc and Cyclin D (Fig. 5C). As a result, compared with mock-treated group, Iacs-miR-30C statistically significantly suppressed the tumor growth in more action than 5-Fu as evidence by the tumor volume curve (Fig. 5D), tumor growth inhibition (TGI) effect (Fig. 5E), tumor photos (Fig. 5F) and tumor weights (Fig. 5G). More importantly, the combination therapy between Iacs-miR-30C and 5-Fu showed a significant increase anti-tumor action in contrast to the monotherapy of Iacs-miR-30C or 5-Fu (Fig. 5D–G), suggesting their synergistic sensitized effect. Moreover, proliferative cells analysis (ki67 staining) and apoptotic cells analysis (TUNEL staining) also supported these results (Figs. 5H and I). Collectively, our data illustrated that Iacs-miR-30C potently suppressed Wnt/β-catenin pathway in vivo and augmented the anti-tumor action of the chemotherapeutic agent 5-FU.

### IacsRNA sensitized tumor immunotherapy

**Fig. 6:**
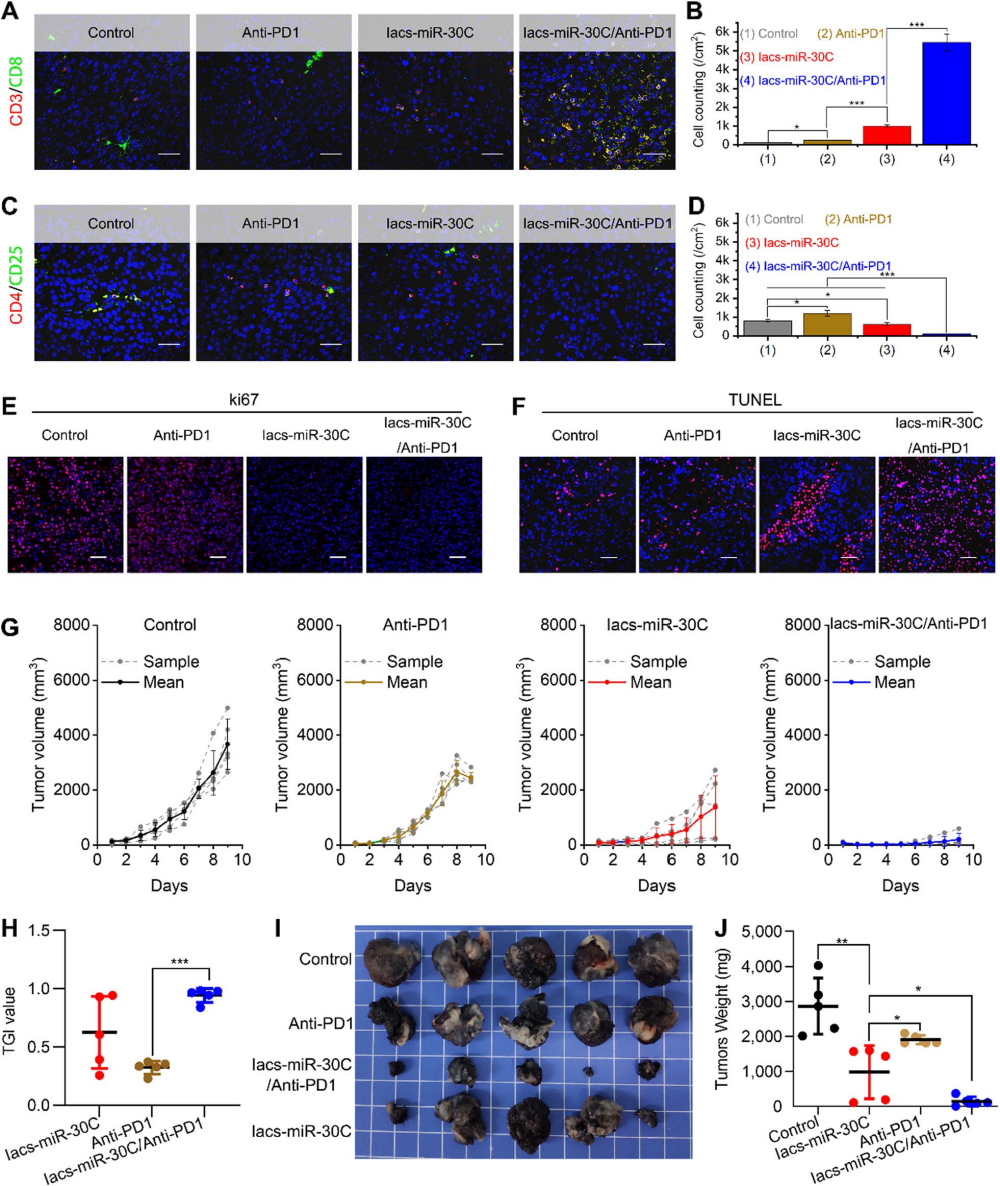
IacsRNA enhanceed anti-cancer activity of PD1 in C57 mice bearing B16F10 tumor. **A**, **B** Immunofluorescence images of CD3^+^/CD8^+^ cells (**A**) and quantification (**B**) in tumor sections from mice with the indicated treatments (scale bar: 50 μm). **C**, **D** Immunofluorescence images of CD4^+^/CD25^+^ cells (**C**) and quantification (**D**) in tumor sections from mice with the indicated treatments (scale bar: 50 μm). **E**, **F** Ki67 (**E**) and TUNEL (**F**) staining in tumor sections from mice with the indicated treatments (scale bar: 50 μm). **G** Growth curves of B16F10 tumors in C57 mice with the indicated treatments (n = 5). **H** Tumor growth inhibition (TGI) with the indicated treatment at day 9. **I**, **J** Representative photographs (**I**) and weight of tumor (**J**) after the indicated treatments. The data were presented as mean ± s.d. Statistical analysis was performed using t test, *, *p* < 0.05; **, *p* < 0.01; ***, *p* < 0.001

Programmed cell death ligand 1 (PD-L1) on tumor cells or tumor derived exosomes binds programmed cell death 1 (PD-1) on T cells and efficiently stimulates cytotoxic CD8^+^T cell malfunctioning and apoptosis, allowing cancer cells to escape from the immune attack [17, 40, 41]. Anti PD-1 antibodies or anti PD-L1 antibodies are thus considered as effective anti-tumor drugs, but its anti-tumor response is always restrained to T cell infiltration. In tumor, Wnt signaling results in T-cell exclusion and resistance to anti-PD-L1 or anti-PD-1 therapy [42]. Thus, we speculated that suppressing Wnt signaling cascade by lacs-miR-30C can sensitized the PD1/PD-L1 immuno-checkpoint blocking therapy. To verify it, we established an immunotherapeutic model in which C57/B6L mice were subcutaneously inoculated with B16F10 cells on the flank and treated with Iacs-miR-30C, anti-PD-1 antibody or Iacs-miR-30C/Anti-PD-1 antibody combo. As shown in Additional file 1: Figures S4 and S5, Iacs-miR-30C suppressed the β-catenin/Wnt signaling cascade in B16F10 tumor as support by the decreased protein level of Bcl9, β-catenin, C-myc and Cyclin D. Not surprisingly, compared with Anti-PD-1 or Iacs-miR-30C monotherapy, the combo treatment resulted in a significantly decreased amounts of regulatory T lymphocyte (CD4^+^/CD25^+^ cells, Fig. 6A, B) in sharp contrast to an increased number of tumor-infiltrating cytotoxic T lymphocyte (CD3^+^/CD8^+^ cells, Fig. 6C, D). The inhibited tumor cell proliferation (Fig. 6E) and the enhanced tumor cell apoptosis (Fig. 6F) also supported the synergistic effect of Iacs-miR-30C on anti-PD-1 therapy. As a result, Iacs-miR-30C/Anti-PD-1 combo therapy led to dramatically increased TGI value (94.3% TGI) as compared to anti-PD-1 therapy (37.5% TGI) or Iacs-miR-30C therapy (62.7% TGI) (Fig. 6G and H), in line with the results of the tumor photos (Fig. 6I) and weights (Fig. 6J). Collectively, these results provided abundant evidences that Iacs-miR-30C treatment allowed overcoming T cell exclusion and amplified anti-tumor effects in anti-PD-1 antibody therapy through suppressing Wnt signaling cascade.

## Discussion

Wnt signaling cascades play a crucial part in embryonic development, tissue homeostasis and stem cells proliferation and differentiation for all animals [43]. The aberrant activation of this signaling, however, underlies multiple human cancers, including but not limited to intestinal cancer, lung cancer, liver cancer and lymphoma [44]. In this case, dysregulated Wnt signaling cascades accumulates the transcriptional activator β-catenin in the cytoplasm and the nucleus, resulting in the promotion of the genes expression for malignant cell proliferation and metastasis. In the process, as a component of the aberrantly activated Wnt signaling pathway, β‑catenin coactivators, including Bcl9, are always overexpressed in a variety of malignancies, and compose a stable complex with β-catenin to increase such malignant gene expression. [45] What’s worse, dysregulated Wnt signaling cascades will reduce the infiltration of chemotherapeutics and T-lymphocytes (T-cell), resulting in the resistance of the chemotherapies and PD-1/PD-L1 checkpoint-blockade Immunotherapies [46–48]. To addressed it, some researches have verified that the suppression of tumor-intrinsic active β-catenin signaling not only can inhibit the tumor progression, but also sensitized the chemotherapy and Immunotherapy ([49–52]). Of note, the present work amply proved these points: Iacs-miR-30C effectively sensitized both potency of 5-Fu in PDX model of colon cancer (Fig. 5) and Anti-PD1 in B16F10 homograft model of melanoma (Fig. 6).

Although there has existed some success for Wnt/β-catenin suppression, many still remain to be done, in especial of tumor specificity. The activated Wnt pathway is a common element in regulating stem cell/progenitor cell renewal and maintenance in noncancerous tissues and organs [53, 54]. As a results, Wnt/β-catenin inhibitor always cause on-targeted toxicity, therefore none of them has yet been approved for clinical application [16]. To address this issue, Bcl9 that is highly expressed in tumors but not in the cells of tumor origin, has received considerable attention [55]. It has been reported that the oncogenic role of Bcl9 can be rescued by siRNA/ShRNA-induced knockdown or the treatment with stapled Bcl9 peptide, both of which attenuated proliferation, metastasis, and resistance to therapy, highlighting the importance of Bcl9 for targeted oncotherapy [56, 57]. Thus, the development Iacs-miR-30C possibly supplies the gap of safe and effective Wnt inhibitor for clinical transformation.

## Conclusion

Herein, a general method through a mild and simple chemistry was established to convert therapeutic miRNA into a stable and bioavailable IacsRNA. Driven by aurophilicity, IacsRNA self-assembled into a spherical nanostructure with the optimized anti-hydrolysis stability and low macrophage uptakes in comparison to conventional miRNA. As a consequence, IacsRNA presented the increased half-life period in circulation and accumulation at tumor sites in comparison to normal miRNA. More importantly, Iacs-miR-30c showed no toxicity of viscera and sanguis system in the 5-time injection dosage of the treatment. Expectedly, Iacs-miR-30c potently suppressed the Wnt signaling pathway in vitro and in vivo, and effectively sensitized both potency of 5-Fu in PDX model of colon cancer and Anti-PD1 in B16F10 homograft model of melanoma. In short, this work amply confirmed the design of IacsRNA as a general and viable strategy of nano-pharmaceutic to concert flimsy therapeutic miRNA into potential drugs. Considering from a broader perspective, the miRNA-initiated infinite coordination self-assembly strategy has distinct advantages in resurrecting nuclear acid therapeutics, probably bringing new inspiration to RNA-derived therapeutics of a great variety of human diseases including cancer.

## Methods

### lacsRNA construction

2 OD mercapto modified miR-30C were dissolved in 5 ml HEPES buffer (50 mM, pH 7.4), following the magnetic stirring (50 ℃, 550 rpm). Next, 500 µl 10 mM HAuCl_4_ was added into this miRNA- containing buffer. After about 10-min stirring, the solution changes from golden yellow to red violet, suggesting the successful synthesis of lacsRNA. After twice centrifugal washing by ultrapure water, IacsRNA can obtain by lyophilization.

### Patient-derived xenografts (PDX) model of colon carcinoma.

At the time of primary tumor reductive surgery, a specimen was cut into about 5 mm pieces and subcutaneously implanted into the fossa iliaca of NOD/SCID mice aged 4 ~ 5 weeks. Genetic mutations in the colon tumor were reassured by whole exome sequencing (WES). Volume of tumor was observed in subsequent days and calculated by the equation: volume = 1/2 length × width^2^. When the tumors reached approximately 100 ± 30 mm^3^, mice were randomly grouped and intravenously injected therapeutic agents or normal saline (NS) every other day.

### B16F10 melanoma model

B16F10 cells were harvested at confluency of near 100% in dish, and pelleted by centrifugation and resuspended in sterile PBS buffer. Next, B16F10 cells (4 × 10^6^ per site) were subcutaneously inoculated into the crotch of immunocompetent C57/B6L mice aged about five weeks. When the tumors reached average volume range from 50 to 100 mm^3^, the mice were randomly divided into different groups (n = 5) and were treated with therapeutic agents or isometric NS. Tumor growth and body weights were observed in subsequent days.

## Supplementary Information

**Additional file 1.** **Figure S1.** Vis-UV spectrum of RNA (miR-30C) and IacsRNA (Iacs-miR-30C). **Figure S2.** Gene set enrichment analysis (GSEA) showing the cell cycle checkpoints and cell cycle mitotic differentially expressed in response to IacsRNA. **Figure S3.** Cell cycle of B16F10 cells after 1-day miR-30C and Iacs-miR-30C treatment at a dosage of 100 nM. **Figure S4.** Representative IF staining for Bcl9 and IHC staining for β-catenin in B16F10 tumors with different treatments. **Figure S5.** Representative IF staining for c-Myc and Cyclin D1 in B16F10 tumors with different treatments. **Table S1.** Hydrodynamic diameter at different pH values measured by DLS. Supplementary experimental section.

## Data Availability

The datasets used and/or analyzed during the current study are available from the corresponding author on reasonable request.

## References

[1] Ganju A, Khan S, Hafeez BB, Behrman SW, Yallapu MM, Chauhan SC, Jaggi M (2017). miRNA nanotherapeutics for cancer. Drug Discov Today.

[2] Zhao XH, Wang P, Liu J, Zheng J, Liu YH, Chen JJ, Xue YX (2015). Gas5 exerts tumor-suppressive functions in human glioma cells by targeting miR-222. Mol Ther.

[3] Wen SY, Lin Y, Yu YQ, Cao SJ, Zhang R, Yang XM, Li J, Zhang YL, Wang YH, Ma MZ (2015). miR-506 acts as a tumor suppressor by directly targeting the hedgehog pathway transcription factor Gli3 in human cervical cancer. Oncogene.

[4] Kai ZS, Pasquinelli AE (2010). MicroRNA assassins: factors that regulate the disappearance of miRNAs. Nat Struct Mol Biol.

[5] Yin H, Kanasty RL, Eltoukhy AA, Vegas AJ, Dorkin JR, Anderson DG (2014). Non-viral vectors for gene-based therapy. Nat Rev Genet.

[6] Morille M, Passirani C, Vonarbourg A, Clavreul A, Benoit JP (2008). Progress in developing cationic vectors for non-viral systemic gene therapy against cancer. Biomaterials.

[7] Pattni BS, Chupin VV, Torchilin VP (2015). New developments in liposomal drug delivery. Chem Rev.

[8] Bozzuto G, Molinari A (2015). Liposomes as nanomedical devices. Int J Nanomed.

[9] Huang SN, Duan SF, Wang J, Bao SJ, Qiu XJ, Li CM, Liu Y, Yan LJ, Zhang ZZ, Hu YR (2016). Folic-acid-mediated functionalized gold nanocages for targeted delivery of anti-miR-181b in combination of gene therapy and photothermal therapy against hepatocellular carcinoma. Adv Funct Mater.

[10] Chen X, Gu S, Chen BF, Shen WL, Yin Z, Xu GW, Hu JJ, Zhu T, Li G, Wan C (2015). Nanoparticle delivery of stable miR-199a-5p agomir improves the osteogenesis of human mesenchymal stem cells via the HIF1a pathway. Biomaterials.

[11] Yu M, Lei B, Gao CB, Yan J, Ma PX (2017). Optimizing surface-engineered ultra-small gold nanoparticles for highly efficient miRNA delivery to enhance osteogenic differentiation of bone mesenchymal stromal cells. Nano Res.

[12] Yu M, Xue YM, Ma PX, Mao C, Lei B (2017). Intrinsic ultrahigh drug/miRNA loading capacity of biodegradable bioactive glass nanoparticles toward highly efficient pharmaceutical delivery. ACS Appl Mater Inter.

[13] Yu M, Yan J, He W, Li C, Ma PX, Lei B (2017). Synthetic θ-defensin antibacterial peptide as a highly efficient nonviral vector for redox-responsive miRNA delivery. Adv Biosyst.

[14] Li HJ, Du JZ, Du XJ, Xu CF, Sun CY, Wang HX, Cao ZT, Yang XZ, Zhu YH, Nie SM, Wang J (2016). Stimuli-responsive clustered nanoparticles for improved tumor penetration and therapeutic efficacy. Proc Natl Acad Sci USA.

[15] Zanganeh S, Hutter G, Spitler R, Lenkov O, Mahmoudi M, Shaw A, Pajarinen JS, Nejadnik H, Goodman S, Moseley M (2016). Iron oxide nanoparticles inhibit tumour growth by inducing pro-inflammatory macrophage polarization in tumour tissues. Nat Nanotechnol.

[16] He W, Yan J, Jiang W, Li S, Qu Y, Niu F, Yan Y, Sui F, Wang S, Zhou Y (2018). Peptide-induced self-assembly of therapeutics into a well-defined nanoshell with tumor-triggered shape and charge switch. Chem Mater.

[17] Yan J, He W, Li X, You W, Liu X, Lin S, Chen J, Zhao Y, Zhang Y, Ji F (2021). Carnosic acid-induced co-self-assembly of metal-peptide complexes into a nanocluster-based framework with tumor-specific accumulation for augmented immunotherapy. Chem Eng J.

[18] Zheng X, Yan J, You W, Li F, Diao J, He W, Yao Y (2021). De novo nano-erythrocyte structurally braced by biomimetic Au(I)-peptide skeleton for MDM2/MDMX predation toward augmented pulmonary adenocarcinoma immunotherapy. Small.

[19] He W, Yan J, Li Y, Yan S, Wang S, Hou P, Lu W (2020). Resurrecting a p53 peptide activator—an enabling nanoengineering strategy for peptide therapeutics. J Control Release.

[20] Yan J, Yao Y, Yan S, Gao R, Lu W, He W (2020). Chiral protein supraparticles for tumor suppression and synergistic immunotherapy: an enabling strategy for bioactive supramolecular chirality construction. Nano Lett.

[21] She J, Li Y, Yan S, Yan Y, Liu D, Li S, Guo Y, Xue Y, Yao Y, Yan J, He W (2020). De novo supraparticle construction by a self-assembled Janus cyclopeptide to tame hydrophilic microRNA and hydrophobic molecule for anti-tumor cocktail therapy and augmented immunity. Chem Eng J.

[22] Barenholz Y (2012). Doxil(R)–the first FDA-approved nano-drug: lessons learned. J Control Release.

[23] Jain RK, Stylianopoulos T (2010). Delivering nanomedicine to solid tumors. Nat Rev Clin Oncol.

[24] Ma B, Niu F, Qu X, He W, Feng C, Wang S, Ouyang Z, Yan J, Wen Y, Xu D (2019). A tetrameric protein scaffold as a nano-carrier of antitumor peptides for cancer therapy. Biomaterials.

[25] Mei H, Cai S, Huang D, Gao H, Cao J, He B (2022). Carrier-free nanodrugs with efficient drug delivery and release for cancer therapy: from intrinsic physicochemical properties to external modification. Bioact Mater.

[26] Karaosmanoglu S, Zhou M, Shi B, Zhang X, Williams GR, Chen X (2021). Carrier-free nanodrugs for safe and effective cancer treatment. J Control Release.

[27] Liu T, Yan J, He C, You W, Ma F, Chang Z, Li Y, Han S, He W, Liu W (2021). A tumor-targeting metal-organic nanoparticle constructed by dynamic combinatorial chemistry toward accurately redressing carcinogenic Wnt cascade. Small.

[28] Imaz I, Rubio-Martinez M, Garcia-Fernandez L, Garcia F, Ruiz-Molina D, Hernando J, Puntes V, Maspoch D (2010). Coordination polymer particles as potential drug delivery systems. Chem Commun.

[29] Luo S, Wang Y, Shen S, Tang P, Liu Z, Zhang S, Wu D (2021). IR780-loaded hyaluronic acid@Gossypol–Fe(III)–EGCG infinite coordination polymer nanoparticles for highly efficient tumor photothermal/coordinated dual drugs synergistic therapy. Adv Funct Mater.

[30] Zhao JJ, Lin JH, Zhu D, Wang XJ, Brooks D, Chen M, Chu ZB, Takada K, Ciccarelli B, Admin S (2014). miR-30-5p functions as a tumor suppressor and novel therapeutic tool by targeting the oncogenic Wnt/beta-catenin/BCL9 pathway. Cancer Res.

[31] Negishi Y, Nobusada K, Tsukuda T (2005). Glutathione-protected gold clusters revisited: bridging the gap between gold (I)− thiolate complexes and thiolate-protected gold nanocrystals. J Am Chem Soc.

[32] Yan J, Ji F, Yan S, You W, He W (2020). A general-purpose nanohybrid fabricated by polymeric Au(I)-peptide precursor to wake the function of peptide therapeutics. Theranostics.

[33] He W, Wang S, Yan J, Qu Y, Jin L, Sui F, Li Y, You W, Yang G, Yang Q (2019). Self-assembly of therapeutic peptide into stimuli-responsive clustered nanohybrids for cancer-targeted therapy. Adv Funct Mater.

[34] Champion JA, Walker A, Mitragotri S (2008). Role of particle size in phagocytosis of polymeric microspheres. Pharm Res.

[35] Bartneck M, Keul HA, Singh S, Czaja K, Bornemann J, Bockstaller M, Moeller M, Zwadlo-Klarwasser G, Groll J (2010). Rapid uptake of gold nanorods by primary human blood phagocytes and immunomodulatory effects of surface chemistry. ACS Nano.

[36] He W, Yan J, Wang L, Lei B, Hou P, Lu W, Ma PX (2019). A lanthanide-peptide-derived bacterium-like nanotheranostic with high tumor-targeting, -imaging and -killing properties. Biomaterials.

[37] Yan J, He W, Yan S, Niu F, Liu T, Ma B, Shao Y, Yan Y, Yang G, Lu W (2018). Self-assembled peptide-lanthanide nanoclusters for safe tumor therapy: overcoming and utilizing biological barriers to peptide drug delivery. ACS Nano.

[38] Yan J, Yan S, Hou P, Lu W, Ma PX, He W, Lei B (2019). A Hierarchical peptide-lanthanide framework to accurately redress intracellular carcinogenic protein-protein interaction. Nano Lett.

[39] Tabata Y, Ikada Y (1988). Macrophage phagocytosis of biodegradable microspheres composed of L-lactic acid/glycolic acid homo- and copolymers. J Biomed Mater Res.

[40] Wang Y, Zhou S, Yang F, Qi X, Wang X, Guan X, Shen C, Duma N, Vera Aguilera J, Chintakuntlawar A (2019). Treatment-related adverse events of PD-1 and PD-L1 inhibitors in clinical trials: a systematic review and meta-analysis. JAMA Oncol.

[41] Liu J, Yan J, Yan S, Wang Y, Zhang R, Hou P, He W, Ji M (2019). Biomimetic and self-assembled nanoclusters targeting β-catenin for potent anticancer therapy and enhanced immunotherapy. Nano Lett.

[42] Feng M, Jin JQ, Xia L, Xiao T, Mei S, Wang X, Huang X, Chen J, Liu M, Chen C (2019). Pharmacological inhibition of β-catenin/BCL9 interaction overcomes resistance to immune checkpoint blockades by modulating Treg cells. Sci Adv.

[43] Moon RT, Kohn AD, De Ferrari GV, Kaykas A (2004). WNT and beta-catenin signalling: diseases and therapies. Nat Rev Genet.

[44] Reya T, Clevers H (2005). Wnt signalling in stem cells and cancer. Nature.

[45] Ling XH, Chen ZY, Luo HW, Liu ZZ, Liang YK, Chen GX, Jiang FN, Zhong WD (2016). BCL9, a coactivator for Wnt/-catenin transcription, is targeted by miR-30c and is associated with prostate cancer progression. Oncol Lett.

[46] Lake RA, Robinson BW (2005). Immunotherapy and chemotherapy—a practical partnership. Nat Rev Cancer.

[47] Chen M, Zhou X, Chen R, Wang J, Ye RD, Wang Y, Wu C, Mahato RI (2019). Nano-carriers for delivery and targeting of active ingredients of Chinese medicine for hepatocellular carcinoma therapy. Mater Today.

[48] Spranger S, Bao R, Gajewski T (2014). Melanoma-intrinsic β-catenin signaling prevents T cell infiltration and anti-tumor immunity. J ImmunoTher Cancer.

[49] Martins-Neves SR, Paiva-Oliveira DI, Wijers-Koster PM, Abrunhosa AJ, Fontes-Ribeiro C, Bovee JVMG, Cleton-Jansen AM, Gomes CMF (2016). Chemotherapy induces sternness in osteosarcoma cells through activation of Wnt/beta-catenin signaling. Cancer Lett.

[50] Wang YX, Liu CZ, Luo M, Zhang ZY, Gong JN, Li JJ, You L, Dong L, Su R, Lin HS (2015). Chemotherapy-induced miRNA-29c/catenin-delta signaling suppresses metastasis in gastric cancer. Cancer Res.

[51] Gattinoni L, Ji Y, Restifo NP (2010). Wnt/beta-catenin signaling in T-cell immunity and cancer immunotherapy. Clin Cancer Res.

[52] Suryawanshi A, Tadagavadi RK, Swafford D, Manicassamy S (2016). Modulation of inflammatory responses by wnt/beta-catenin signaling in dendritic cells: a novel immunotherapy target for autoimmunity and cancer. Front Immunol.

[53] Merrill BJ (2012). Wnt pathway regulation of embryonic stem cell self-renewal. Cold Spring Harb Perspect Biol.

[54] Xiao L, Yuan X, Sharkis SJ (2006). Activin A maintains self-renewal and regulates fibroblast growth factor, Wnt, and bone morphogenic protein pathways in human embryonic stem cells. Stem Cells.

[55] Takada K, Zhu D, Bird GH, Sukhdeo K, Zhao JJ, Mani M, Lemieux M, Carrasco DE, Ryan J, Horst D (2012). Targeted disruption of the BCL9/beta-catenin complex inhibits oncogenic Wnt signaling. Sci Transl Med..

[56] Deka J, Wiedemann N, Anderle P, Murphy-Seiler F, Bultinck J, Eyckerman S, Stehle JC, Andre S, Vilain N, Zilian O (2010). Bcl9/Bcl9l are critical for Wnt-mediated regulation of stem cell traits in colon epithelium and adenocarcinomas. Cancer Res.

[57] Brembeck FH, Wiese M, Zatula N, Grigoryan T, Dai YY, Fritzmann J, Birchmeier W (2011). BCL9-2 Promotes early stages of intestinal tumor progression. Gastroenterology.

