# Supplementary material for: Turing miRNA into infinite coordination supermolecule: a general and enabling nanoengineering strategy for resurrecting nuclear acid therapeutics
Source: J Nanobiotechnology. 2022 Jan 4;20:10. doi: 10.1186/s12951-021-01212-9 (PMC8725389; doi:10.1186/s12951-021-01212-9)
Supplement: Supplementary file 1 — Additional file 1. Figure S1. Vis-UV spectrum of RNA (miR-30C) and IacsRNA (Iacs-miR-30C). Figure S2. Gene set enrichment analysis (GSEA) showing the cell cycle checkpoints and cell cycle mitotic differentially expressed in response to IacsRNA. Figure S3. Cell cycle of B16F10 cells after 1-day miR-30C and Iacs-miR-30C treatment at a dosage of 100 nM. Figure S4. Representative IF staining for Bcl9 and IHC staining for β-catenin in B16F10 tumors with different treatments. Figure S5. Representative IF staining for c-Myc and Cyclin D1 in B16F10 tumors with different treatments. Table S1. Hydrodynamic diameter at different pH values measured by DLS. Supplementary experimental section. [file 12951_2021_1212_MOESM1_ESM.docx]

**Additional Materials**

**Turing miRNA into infinite coordination supermolecule:**

**a general and enabling nanoengineering strategy for resurrecting** **nuclear acid therapeutics**

**Liya Li^1, †^, Wangxiao He^2, 1,*,†^, Weiming You^3^, Jin Yan^3,*^ and Wenjia Liu^1, *^**

^1^Institute for Stem Cell & Regenerative Medicine, The Second Affiliated Hospital of Xi’an Jiaotong University, Xi’an 710004, China

^2^Department of Medical Oncology and Department of Talent Highland, The First Affiliated Hospital of Xi’an Jiaotong University, Xi’an 710061, PR. China.

^3^National & Local Joint Engineering Research Center of Biodiagnosis and Biotherapy, The Second Affiliated Hospital of Xi'an Jiaotong University, Xi'an, 710004, PR. China.

^†^ These authors contributed equally.

^*^ **Corresponding authors**:

Email (W. He): [hewangxiao5366@xjtu.edu.cn](mailto:hewangxiao5366@xjtu.edu.cn);

Email (J. Yan): yanjin19920602@xjtu.edu.cn;

Email (W. Liu): wenjialiu@xjtu.edu.cn.

**Additional Figures**





**Figure S1.** Vis-UV spectrum of RNA (miR-30C) and IacsRNA (Iacs-miR-30C)


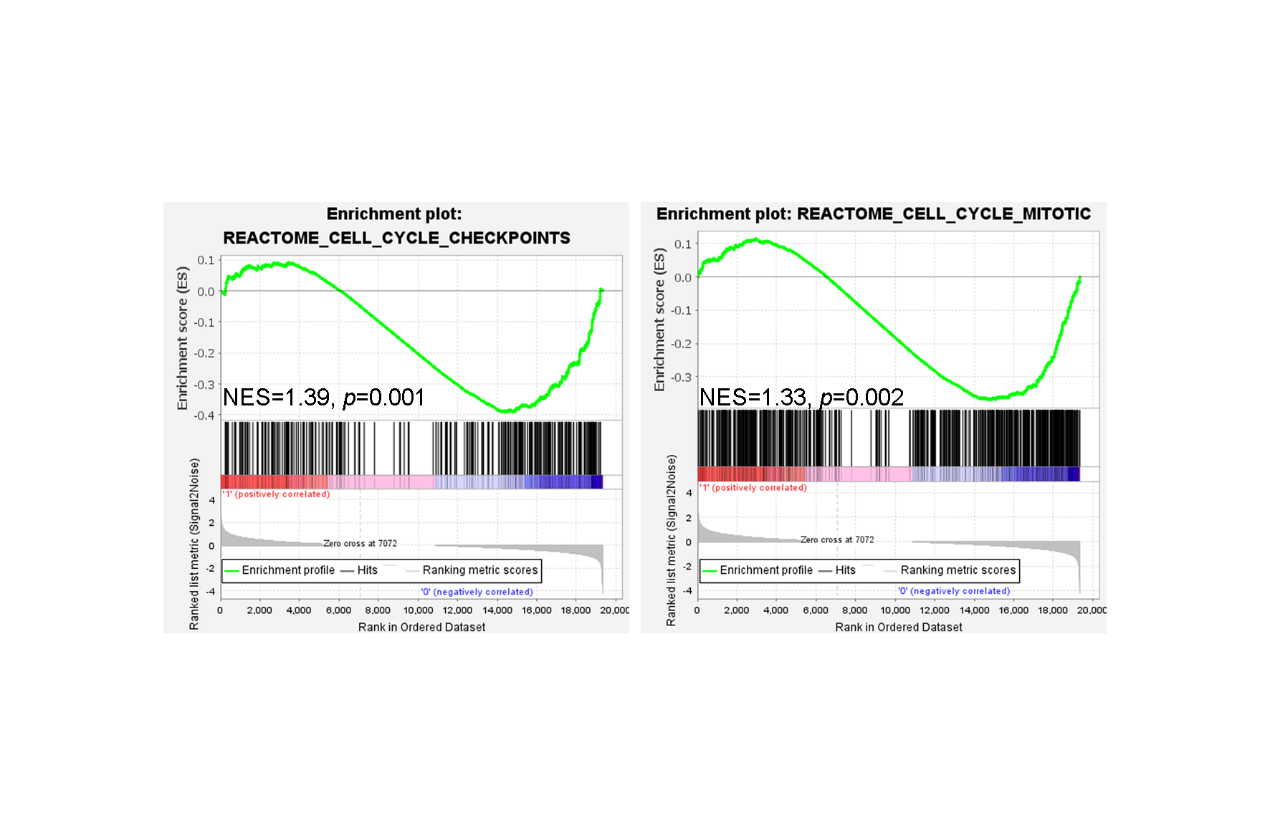


**Figure S2.** Gene set enrichment analysis (GSEA) showing the cell cycle checkpoints and cell cycle mitotic differentially expressed in response to IacsRNA.


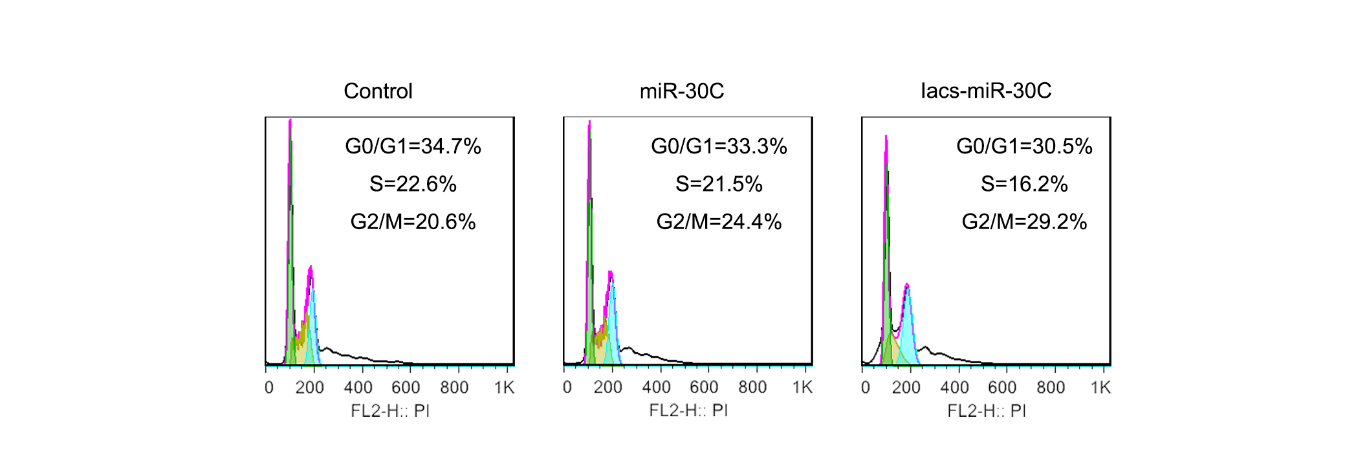


**Figure S3.** Cell cycle of B16F10 cells after 1-day miR-30C and Iacs-miR-30C treatment at a dosage of 100 nM.


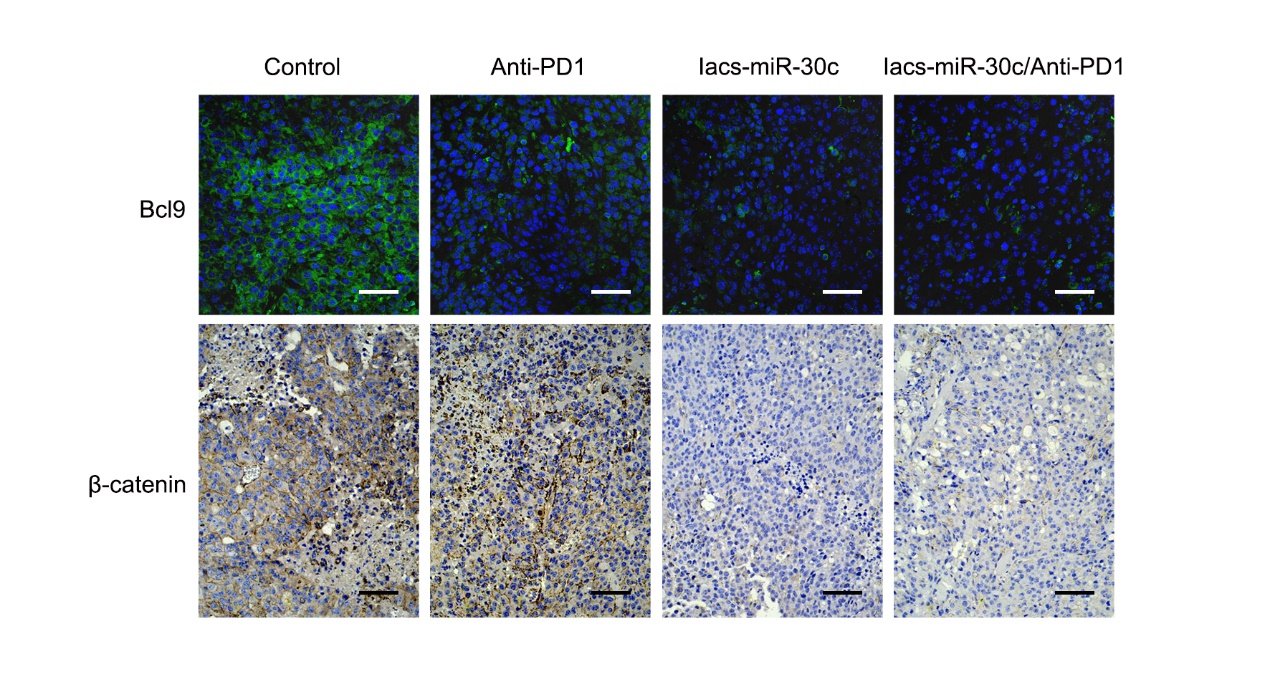


**Figure S4.** Representative IF staining for Bcl9 and IHC staining for β-catenin in B16F10 tumors with different treatments.


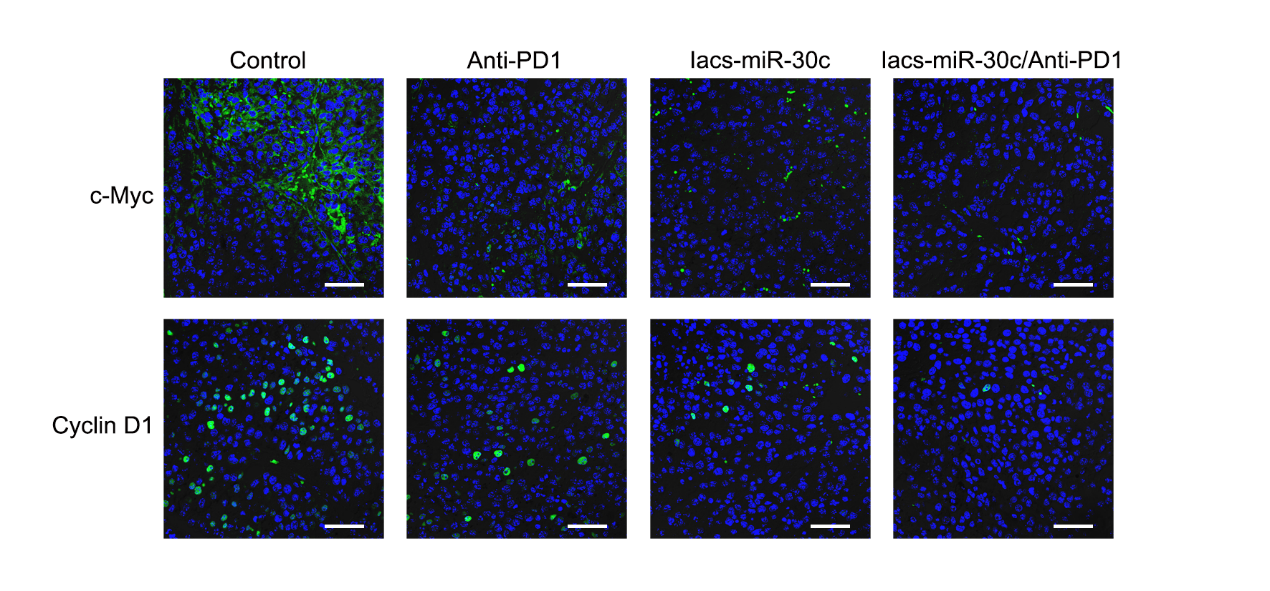


**Figure S5.** Representative IF staining for c-Myc and Cyclin D1 in B16F10 tumors with different treatments.

**Additional Tables**

| Table S1. Hydrodynamic diameter at different pH values measured by DLS | | |
| --- | --- | --- |
| pH | Hydrodynamic diameter (nm) | PDI |
| 5 | 69.9 | 0.269 |
| 6 | 58.9 | 0.278 |
| 7 | 45.8 | 0.365 |
| 7.4 | 38.7 | 0.265 |
| 8 | 30.5 | 0.210 |

**Additional experimental section**

**General remarks**

Synthetic miRNAs were obtained from GenePharma (China). All other chemicals used in this study were purchased from Sigma-Aldrich unless otherwise specified. Acetonitrile and water (HPLC grade) were purchased from Fisher Scientific Ltd. All products were used as received without further purification.

**Physicochemical properties of lacsRNA**

The morphology of IacsRNA was observed on high-resolution transmission electron microscope (HRTEM) (ThermoFisher Talos-F200X) operated at 200kV. The hydrodynamic size distribution (1 mg/mL in PBS, 1 mL) was obtained from the dynamic light scattering (DLS) measurement (Malvern Zetasizer Nano ZS system). For Zeta potential measurement, the nanoparticles (1 mg/mL, 1 mL) were incubated with PBS at different pH at 37 ºC for 30 min, and measured by dynamic light scattering (DLS).

**Stability of IacsRNA**

Vis-UV absorption spectra were obtained from Shimadzu 3000 spectrophotometer. RNA stability was obtained via performing agarose gel electrophoresis of 250ng miR-30c or IacsRNA containing equal amount of miR-30c incubated in 25% bovine serum sterile PBS at room temperature. The reaction was terminated via adding 2-mercaptoethanol (2%) and RNase inhibitor (40U/100µl) (Beyotime, China) at multiple time points and the samples were stored at -20℃. IacsRNA sample underwent 10,000 rpm centrifugation at 4℃ to collect miR-30C in the supernatant.

**Cell culture and viability analysis**

Mouse B16F10 cell were purchased by ATCC, and maintained in RPMI1640 medium with 10% FBS. Human HCT116 cell line were also purchased by ATCC, and maintained in McCoy’s 5A medium with 10% FBS. Cells were first serum starved for 12 h, and then treated with miR-30C and IacsRNA at a dosage of 100 nM for other 24 h. Next, cells were harvested, washed twice in PBS, and fixed in 70% ethanol on ice for at least 30 min. After that, cells were stained with propidium iodide (PI) solution (50 μg/ml PI, 50 μg/ml RNase A, 0.1% Triton-X, 0.1mM EDTA). Cell cycle distributions were then analyzed based on DNA contents by a flow cytometer (BD Biosciences, NJ).

**Cellular uptake of PMI-Au SNH**

Cellular uptake of miR-30C and IacsRNA was detected using flow cytometer (BD Biosciences, NJ). Cy3 was firstly labeled to the 5’-terminal of miR-30C. Similarly, ^Cy3^IacsRNA was prepared as above. First, ^Cy3^IacsRNA and ^Cy3^miR-30C were re-dispersed in culture medium at a concentration of 2μM, and B16F10 melanoma cells and RAW264.7 macrophages were cultured in corresponding growth medium for 24 h. The medium was then replaced with the conditional medium containing ^Cy3^IacsRNA and ^Cy3^miR-30C at pH 7.4, and further cultured at 37°C for 6 h. Flow cytometry analysis were then carried out after washing the cells with PBS twice to remove the excess ^Cy3^IacsRNA and ^Cy3^miR-30C.

***In vivo* bio-distribution analysis**

B16F10 cells (4 × 10^6^ cells/site) were implanted subcutaneously into hip of five-weeks-old C57/B6L mice. Four weeks after cell inoculation, tumor-bearing mice were injected with 200 μL Cy3-labelled IacsRNA and miR-30C. IVIS Spectrum *In Vivo* Imaging System was then used to take the *ex vivo* images of mice. Light with a wavelength at 490 nm was used as the excitation source. In addition, mice were humanely killed at predetermined times, and the heart, liver, spleen, lung, kidney and tumor were immediately collected from each mouse. The fluorescence intensity in all organs was further analyzed by the IVIS Spectrum *In Vivo* Imaging System.

**Quantification of nanoparticle accumulation and pharmacokinetics using ICP-MS**

The gold content inside any organ can be measured using ICP-MS. B16F10 tumors or organs were resected, weighed, and placed in 50 ml Falcon tubes. They were then digested with 2 ml of nitric acid and 0.5 ml of hydrochloric acid at 70–80 °C overnight. The tissues appeared digested and dissolved. The samples were diluted to 50 ml with deionized water and then filtered with 0.22 µm PES filters (Millipore) using a 10 ml syringe. The filtered digest was then processed using ICP-MS and analyzed using a standard curve derived from stock with a known quantity of gold.

**RNA-sequencing (RNA-seq) and analysis**

RNA was isolated from HCT116 cells and B16F10 cells after 24 hours of treatment with IacsRNA or PBS using the Direct-zol RNA MiniPrep Kit (Zymo Research). Short-read sequencing method was used to capture high-quality, quantitative expression levels. Briefly, the core steps included RNA extraction, mRNA fragmentation(300bp), cDNA synthesis, library construction (NEBNext® Ultra RNA Library Prep Kit for Illumina®), PCR amplification, library quality control (Agilent 2100 Bioanalyzer), sequencing (Illumina HiSeq X Ten) and analysis. Three biological replicates were sequenced for each sample. Heat maps and Gene Expression Enrichment Analysis were generated using the Qlucore Omics Explorer 3.2. Pathway analysis was performed using Ingenuity Pathway Analysis (IPA) software.

**Mouse study**

All mice were purchased from the Laboratory Animal Center of Xi’an Jiaotong University. Animals were housed under standard specific pathogen-free conditions with standard chow and typical light/dark cycles. All experimental procedures involving animals were conducted in accordance with Institution Guidelines and were approved by the medical ethics committee of Xi’an Jiaotong University (XJTU1AF2020LSK-276).

**Immunohistochemical (IHC) staining**

Sections were cut at 4 µm thickness, deparaffinized and rehydrated. Endogenous peroxidase activity was blocked with hydrogen peroxide/methanol, and antigen retrieval was performed in a pH 9.0 TE (Tris-EDTA) buffer by autoclave for 10 min. Frozen sections were cut at 10µm thickness, washed for 5min for three times by PBS and incubated with 5% BSA for 1h at room temperature. The resultant tissue sections were then incubated with the antibodies against β-catenin (Abcam, ab32572), Cyclin D1 (CST55506), c-Myc (Abcam, ab32072), Bcl9 (Abcam, ab37305) and Ki-67 (Abcam, ab15580) at 4°C overnight. For frozen sections, immunofluorescence signals were obtained from confocal microscope (Leica TCS SP8 STED 3X). For paraffin embedded sections, after incubation with labeled streptavidin-biotin (LSAB) complex for 15 min, the slides were stained and visualized by using the iView DAB detection system (ZSGB-BIO, P.R. China). Tumor tissues were manually counted in six fields at 400 × original magnification for further statistical analysis.

**Double immunofluorescence staining of CD3^+^/CD8^+^ T-cells and CD4^+^/CD25^+^ Treg Cells**

The tumor tissues were fixed in 4% paraformaldehyde for 24 h at 4 °C, embedded in tissue-freezing medium (Leica), and sectioned into 4-μm sections. For the immunofluorescence assay, heat-induced antigen retrieval was performed with sodium citrate buffer (10 mmol/L sodium citrate, 0.05% Tween 20, pH 6.0) before the bone sections were blocked with 10% normal serum containing 1% bovine serum albumin (BSA) in Tris-buffered saline and Tween 20 (TBST) (pH 7.6) for 2 h at room temperature; then, the sections were incubated overnight at 4 °C with primary antibodies comprised a mixture of two antibodies: CD3 (Proteintech, 17617-1)/CD8 (Abcam, ab22378), and CD4 (Abcam, ab183685) /CD25 (Biolegend, 101902). For secondary antibodies, the mixture of Alexa Fluor Cy3-conjugated goat-anti-mouse IgG and Alexa Fluor 488-conjugated goat-anti rabbit IgG were used in this study. Tumor tissues that showed double staining in the immunofluorescence procedure were manually counted in six fields at 400× original magnification.

**Toxicity studies**

To assess potential toxicities of repeatedly injecting IacsRNA, which was 5 times the therapeutic concentration. We monitored body weight of all mice over the course of treatment and measured hematological indexes as well as organ function indexes after 9 day treatment. Control mice were implanted with xenograft tumor, but did not receive any treatment. Forty-eight hours after the final infusion, mice were anesthetized with 2% isoflurane, and blood was collected for complete blood count (CBC) determinations, including a red blood cell (RBC), a white blood cell (WBC) count, lymphocyte (LYMPH), Neutrophil (NEUT), Platelets (PLT) and Hemoglobin (HGB). Besides, serum aspartate transaminase (ALT), alanine aminotransferase (AST), blood urea nitrogen (BUN) and creatinine (CRE) were measured by using quantitative enzyme-linked immunosorbent assay (ELISA) kits according to the manufacturer’s instructions. Animals were then euthanized with pentobarbitone to retrieve organs, which were washed with deionized water before fixation in 4% paraformaldehyde. The tissues were processed routinely, and sections were stained with haematoxilin and eosin (H&E).

**Statistical analysis.**

Statistical analyses were performed using two-sided Student's t-test or ANOVA. P <0.05 was considered significant. Data were expressed as mean ± s.d..
